# Extracellular vesicles in the rhizosphere: targets to improve nutrient use efficiency of crops?

**DOI:** 10.3389/fpls.2025.1681793

**Published:** 2025-10-09

**Authors:** Ivan A. Paponov, Stefanie Schulz, Michael Schloter, Ana Conesa, Yehoram Leshem

**Affiliations:** ^1^ Department of Food Science, Aarhus University, Aarhus, Denmark; ^2^ Helmholtz Munich, Research Unit for Comparative Microbiome Analysis, Oberschleissheim, Germany; ^3^ Institute for Integrative Systems Biology (I2SysBio), Spanish National Research Council (CSIC), Paterna, Spain; ^4^ Department of Plant Sciences, MIGAL—Galilee Research Institute, Kiryat-Shmona, Israel; ^5^ Department of Biotechnology, Faculty of Sciences and Technology, Tel Hai College, Upper Galilee, Israel

**Keywords:** extracellular vesicles, nutrient use efficiency, rhizosphere, nitrogen cycling, plant –microbe interactions, root exudates, microbial gene regulation, plant breeding targets

## Abstract

Nutrient use efficiency (NUE) is central to sustainable agriculture, yet major crops such as wheat or barley typically take up only about half of applied fertilizer. The rest is lost through leaching or gaseous emissions, contributing to environmental pollution and climate change. Root exudates play a key role in shaping microbial communities and their functions at the plant–soil interface, catalyzing nutrient mobilization, immobilization, and uptake. Whereas most studies in the past focused on sugars, amino acids, and organic acids excreted by roots, recent evidence highlights extracellular vesicles (EVs) as specialized carriers of proteins, metabolites, and small RNAs (sRNAs) that regulate microbial communities in the rhizosphere. Proteomic studies show that plant EVs contain nutrient transporters, proton ATPases, and aquaporins in their membranes. Once secreted, these vesicles may buffer ions, acidify the microenvironment, or send signals to microbes. Here we discuss the potential of EVs to influence microbes driving crop NUE. We show that EVs carry sRNAs that regulate microbial genes involved in nitrogen cycling, and that plant miRNAs control internal responses to nutrient status. Together, these mechanisms may allow plants to align internal nutrient demand with rhizosphere processes and reduce nitrogen losses from soil. Identifying EV cargo that enhances microbial nutrient turnover or minimizes nutrient losses could guide future breeding. Crop genotypes selected for optimized EV secretion may shape beneficial microbial communities, leading to higher NUE, reduced fertilizer dependence, and lower N_2_O emissions. Therefore, EV-mediated signaling may be considered a promising new breeding target for sustainable crop improvement.

## Introduction

Root exudates play a crucial role in shaping plant–microbe interactions in the rhizosphere. These exudates consist of a diverse range of compounds — from primary metabolites such as carbohydrates, amino acids, and organic acids to specialized molecules including secondary metabolites, proteins, volatile organic compounds (VOCs), and small RNAs (sRNAs) (Badri and Vivanco, 2009; Lee Díaz et al., 2022; Ma et al., 2022; Middleton et al., 2024). Their functions are extensive, involving nutrient mobilization, beneficial microbe recruitment, pathogen defense mediation, and interplant communication (Ma et al., 2022). Early research primarily focused on the role of simple sugars, amino and organic acids, and secondary metabolites, followed by proteins such as enzymes (Badri and Vivanco, 2009). More recently, VOCs have been shown to mediate signaling both below- and aboveground (Lee Díaz et al., 2022), while sRNAs—including microRNAs (miRNAs) as one important class—have emerged as specific regulatory molecules targeting gene expression in both plants and microbes (Middleton et al., 2024).

The excretion of plant-derived metabolites by passive diffusion is a well-established mechanism for many primary metabolites, including VOCs (Canarini et al., 2019). In addition, more complex mechanisms of exudation have been observed for signaling molecules, such as phytohormones, which include transporter-mediated transport and, in some cases, secretion via extracellular vesicles (EVs) (Kretzschmar et al., 2012; Mellor et al., 2022). This is particularly relevant for bioactive molecules that require protection from degradation or tight regulation, including sRNAs and certain secondary metabolites.

In animals and humans, EVs deliver their cargo via multiple pathways, including clathrin- or caveolin-mediated endocytosis, macropinocytosis, phagocytosis, lipid raft–associated uptake, and in some cases direct membrane fusion (Mulcahy et al., 2014). These mechanisms allow EVs to efficiently transport sensitive regulatory molecules across extracellular environments and into recipient cells. In plants, however, the rigid nature of cell wall creates a physical barrier to EV release. Evidence suggests that local cell wall remodeling or loosening—potentially triggered by stress or developmental cues—may facilitate the passage of EVs into the apoplast. Proteomic analyses have shown that plant EVs contain cell wall-modifying enzymes, such as glucanases and peroxidases, indicating that EVs may contribute to loosening the cell wall during secretion and transit (Stotz et al., 2022).

Building on this, here we explore the potential of EVs to transport specific regulatory molecules—such as sRNAs and secondary metabolites—that modulate microbial interactions at the plant–soil interface. We specifically highlight their potential role in enhancing microbial nutrient mobilization and, in turn, improving plant nutrient uptake and nutrient use efficiency.

## The role of the rhizosphere microbiome in the nutrient use efficiency of plants

The rhizosphere microbiome critically regulates plant nutrient use efficiency (NUE) through processes such as nutrient mineralization, solubilization, transport modulation, and hormonal signaling. While nitrogen (N) fixation plays a role primarily in legumes, a broader range of mechanisms involving microbial taxa, such as *Bacillus*, *Pseudomonas*, *Streptomyces*, and arbuscular mycorrhizal fungi (AMF) like *Rhizophagus irregularis* are more universally important in enhancing NUE (Kuzyakov and Razavi, 2019).

Nitrogen mineralization—mediated by saprotrophic bacteria and fungi such as *Streptomyces* spp. and *Trichoderma harzianum*—involves the enzymatic degradation of organic matter, releasing ammonium (NH^4+^) through protease and urease activity (Lazicki et al., 2020). This process governs the rate of N turnover and its synchrony with plant demand for nutrients. In parallel, ammonia-oxidizing bacteria (e.g., *Nitrosospira*) and archaea (e.g., *Nitrososphaera*) mediate nitrification, influencing nitrate (NO_3_^-^) availability and root uptake dynamics (Prosser and Nicol, 2012).

Phosphorus (P) availability is enhanced by phosphate-solubilizing microorganisms (PSMs), notably *Bacillus megaterium* and *Pseudomonas fluorescens*, which secrete organic acids such as gluconic and citric acid that chelate cations and release inorganic phosphate from mineral complexes (Rodrıíguez and Fraga, 1999). AMF such as *Rhizophagus irregularis* form extensive extraradical hyphae that acquire orthophosphate beyond the root depletion zone and transfer it to the host via the mycorrhizal phosphate transporter pathway (Smith and Smith, 2011). While *R. irregularis* is most extensively studied, other species such as *Funneliformis mosseae*, *Claroideoglomus etunicat*um, and *Glomus versiforme* have also been shown to enhance nutrient uptake and soil stability, sometimes with crop- or environment-specific advantages (Shukla et al., 2025).

Microbial modulation of root architecture is another key mechanism. Plant growth-promoting rhizobacteria (PGPR), including *Azospirillum brasilense* and *Pseudomonas putida*, synthesize auxins (notably indole-3-acetic acid) that increase lateral root proliferation and root hair density, expanding the root surface area for nutrient uptake (Spaepen et al., 2007). Additionally, ACC deaminase–producing microbes such as *Pseudomonas fluorescens* lower ethylene levels under nutrient stress, thereby promoting root elongation and nutrient -foraging efficiency (Glick, 2014).

Recent metagenomic studies highlight how plant genotypes influence the recruitment of nutrient-cycling microbes. For example, *NRT1.1B*-mediated NO_3_^-^ signaling in rice modulates root microbiota composition to favor taxa involved in N cycling, thus enhancing NUE (Ju et al., 2024).

Importantly, emerging evidence shows that EVs provide an additional regulatory layer in the rhizosphere. In plants, it has been demonstrated that EVs mediate cross-kingdom transfer of small RNAs, highlighting their capacity for direct molecular communication with microbes (Cai et al., 2018). Plant EVs also selectively package defined sRNAs, including miRNAs, siRNAs, and a distinct class of 10–17 nt “tiny RNAs,” indicating active sorting with signaling potential (Baldrich et al., 2019). Although their direct roles in nutrient cycling remain unclear, such cargo suggests EVs may integrate plant signals with microbial functions relevant for NUE. In line with this, proteomic studies of legume peribacteroid EVs found that ~10% of proteins are linked to RNA metabolism, including DEAD/DEAH-box helicases, pointing to a possible role of EV-mediated RNA transfer in early symbiosis (Ayala-García et al., 2025).

## Extracellular vesicles in the rhizosphere: classical view of plant immunity and pathogen defense

Plants actively release EVs from their roots into the surrounding environment, with these vesicles typically ranging in size from 50 to 100 nm (Palma et al., 2020). EVs contain a variety of bioactive molecules, including proteins, lipids, metabolites, and RNAs, which influence plant–microbe interactions (Ambrosone et al., 2023). Recent evidence demonstrates that EVs mediate cross-kingdom RNA interference (RNAi), transporting plant-derived small RNAs (sRNAs) into fungal cells to silence virulence-related genes (Cai et al., 2018). Thus, EVs potentially represent key vehicles for regulating microbial communities in the rhizosphere.

Much of our current understanding of EVs comes from plant immunity research. Studies have demonstrated that EVs are not only loaded with diverse cargo, including sRNAs and RNA-binding proteins such as AGO1 and RH37, but are also actively internalized by fungal and plant cells (Wang et al., 2024). For example, EVs isolated from Arabidopsis during systemic acquired resistance (SAR) activated immune gene expression and suppressed fungal spore formation. These findings suggest that EVs are not merely passive carriers but also act as active signaling agents, potentially amplifying systemic and cross-kingdom communication.

Emerging evidence links EV-mediated miRNA signaling with both plant nutrition and microbial disease suppression. Liu et al. demonstrated that organic fertilizers, compared to inorganic, chemical fertilizers, significantly enhanced the secretion of specific root miRNAs (sly-miR159 and sly-miR319c-3p) in tomato (Liu et al., 2025). These miRNAs, secreted via exosome-like EVs, contributed to the suppression of the soil - borne pathogen *Ralstonia solanacearum*. Notably, sly-miR159 also promoted beneficial microbes including members of the genera *Streptomyces* and *Bacillus*.

Although EVs have been isolated from various plant tissues —including leaves, roots, pollen, stigma exudates, and seeds— their functions in plant defense and interactions with microbes are only beginning to be fully elucidated (Ambastha et al., 2023; Cai et al., 2023; Suanno et al., 2023). In the context of nitrogen cycling, EVs could act as messengers that regulate microbial activity, potentially influencing the abundance and activity of nitrogen-transforming bacteria. However, no studies have yet quantified the extent to which EVs contribute to nutrient cycling, making this an exciting avenue for future research.

## The role of extracellular vesicles for NUE of crops

Plant EV proteomic studies have detected numerous transporters (e.g., for NO_3_^-^, phosphate, and NH^4+^), proton ATPases, and aquaporins in root-secreted EV samples (Cai et al., 2018), supporting the idea that EVs may play a role in NUE of crops. Intensive studies have focused mainly for legumes and their respective symbionts. Root-exuded flavonoids, delivered via ABC transporters, initiate symbiosis by inducing bacterial nodulation genes (Sugiyama et al., 2007).

A recent study showed that legume roots release EVs containing small RNAs (sRNAs) and peptides that may regulate nitrogen-fixing symbiosis (Ayala-García et al., 2025). Proteomic analyses of EVs from *Lotus burttii* and *Phaseolus vulgaris* revealed that approximately 10% of EV-associated proteins are involved in RNA metabolism, including DEAD/DEAH-box RNA helicases linked to sRNA processing. These findings, along with evidence of inter-kingdom RNA interference—where plants use EVs to deliver sRNAs to other organisms—suggest that EV-mediated sRNA transfer may modulate rhizobial gene expression during early symbiosis (Ayala-García et al., 2025).

In addition to their role in nutrient signaling within the plant, several miRNAs, including miR160, miR167, miR169, miR172, and miR396, have been shown to regulate nodule formation and *Rhizobium* infection in legumes (Yousuf et al., 2021). This underscores their dual role in both nutrient acquisition and plant–microbe communication. The packaging of such miRNAs into EVs may represent a sophisticated mechanism to coordinate microbial symbiosis with host nutrient status. Identifying EV-associated sRNAs enhancing symbiotic efficiency could directly inform crop improvement strategies.

Although most mechanistic research has focused on symbiotic systems, similar EV-mediated processes are predicted to operate in non-legume rhizospheres. In these systems, EVs derived from rhizobacteria have been shown to encapsulate hydrolytic enzymes—such as phosphatases and proteases—that catalyze mineralization of organic phosphorus and nitrogen near root surfaces, potentially elevating localized pools of orthophosphate and NH^4+^ in alignment with root uptake zones (microbial extracellular enzyme dynamics; rhizosphere enzyme-turnover studies) (Tian et al., 2020). EV cargo may also include siderophores and redox-active molecules capable of chelating iron and mobilizing mineral-bound phosphorus, akin to phosphate-solubilizing microbial processes identified for a number of strains from the genera *Bacillus* and *Pseudomonas* (Suleimanova et al., 2023). Although direct miRNA regulation of such groups mediated by plant -derived EVs remains unproven, modulation through EV-induced changes in microbial consortia structure is plausible and warrants further investigation.

In addition, plants such as sorghum, rice, maize, *Brachiaria* grasses, and certain legumes release diverse root -exudate metabolites—terpenoids, phenylpropanoids, benzoxazinoids, quinones—that function as biological nitrification inhibitors (BNIs), suppressing activity of ammonia-oxidizing bacteria (AOB) and archaea (AOA), thereby slowing conversion of NH^4+^ into NO_3_^-^. For instance, syringic acid from rice roots strongly inhibited nitrification and N_2_O emissions in acidic paddy soils by reducing both AOB and AOA abundances (Lu et al., 2022). In maize, hydrophobic compounds such as the benzoxazinoid HDMBOA and a novel quinone dubbed “zeanone” were identified in root exudates, with half -maximal inhibitory concentrations (ED_50_) in low-micromolar range against *Nitrosomonas europaea* and other AOB (Otaka et al., 2022). Overall mechanistic modelling shows that BNI release from roots reduces net nitrogen loss and, under most conditions, improves plant N uptake (Kuppe and Postma, 2024), particularly if root secreted EVs also carry the respective transporters.

Beyond BNIs, recent split−root experiments with *Artemisia annua* have shown that heterogeneous NO_3_^-^ and phosphate supply markedly enhances the exudation of the secondary metabolite artemisinin from nutrient−deficient root sectors, whereas homogeneous deficiency does not (Paponov et al., 2023). This indicates that a combination of local and systemic nutrient signals is required to trigger certain secondary metabolites in the rhizosphere. Although artemisinin does not directly mobilize N or P, such shifts in exudate composition may indirectly affect nutrient cycling by shaping microbial community structure and activity, including organisms involved in nutrient transformations. The response was both species− and compound−specific, as *Hypericum perforatum* did not alter exudation of its secondary metabolites under similar conditions.

## Conclusion and future perspectives

**Figure 1:**
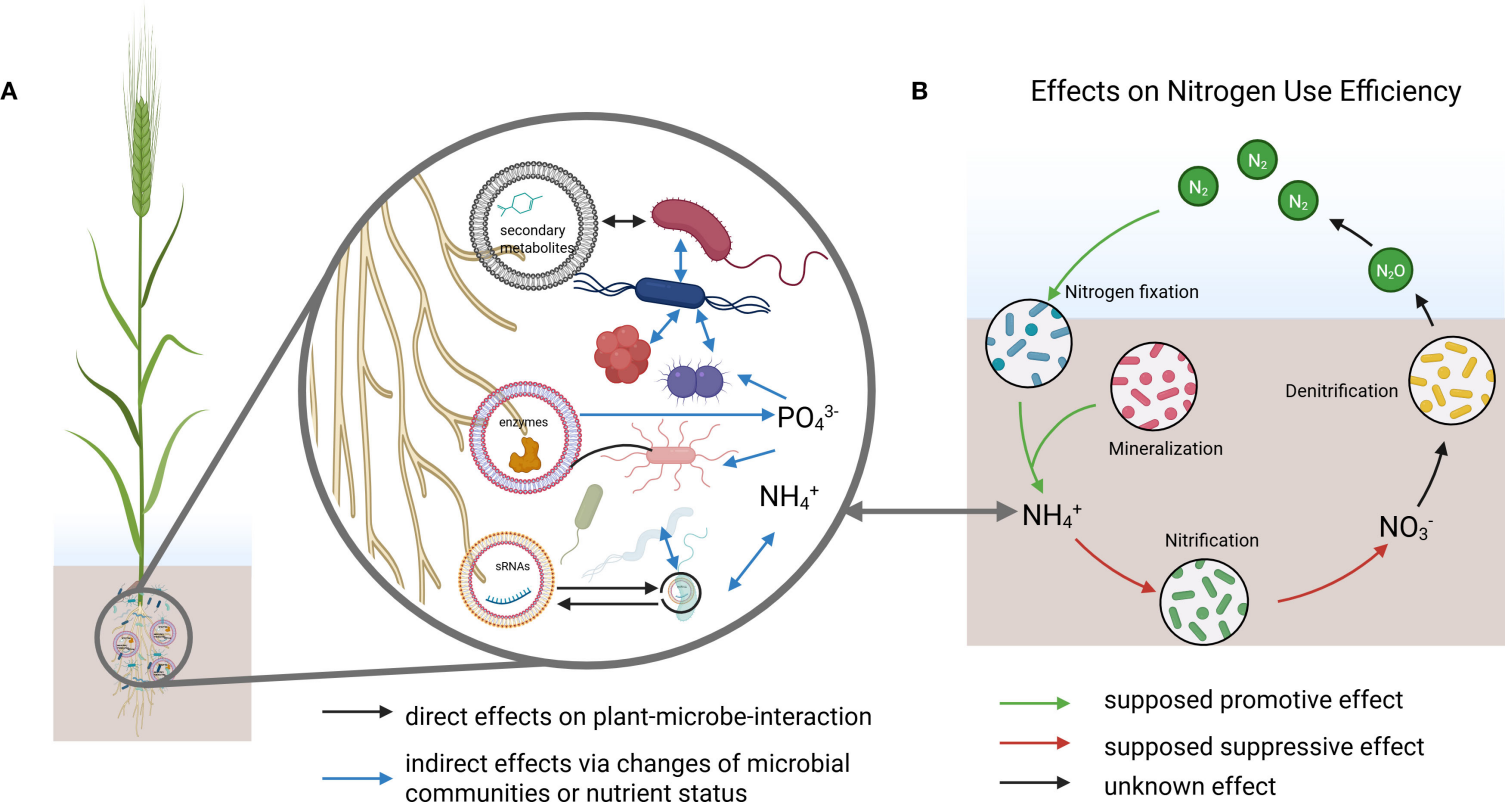
**(A)** Conceptual model of rhizosphere interactions where extracellular vesicles (EVs) deliver secondary metabolites, proteins, and small RNAs (sRNAs) to diverse microorganisms. Arrows indicate direct effects on plant–microbe interactions and indirect effects via shifts in microbial community composition or nutrient status. **(B)** Proposed effects of EV-mediated interactions on nitrogen use efficiency (NUE), linking biological nitrogen fixation, mineralization, nitrification, and denitrification processes. NH^4+^ from **(B)** is connected to NH^4+^ shown in **(A)**. Primary metabolites (e.g., sugars, amino acids, organic acids) are not shown, as their exudation is largely independent from EV release and their general roles in the rhizosphere are well established, which is outside the specific focus of this paper. *Created in BioRender. Schulz, S. (2025)* https://BioRender.com/v6i8jao.

EV-mediated signaling in the rhizosphere is emerging as a potentially critical component in shaping microbial communities that regulate nitrogen cycling. The transport of microRNAs (miRNAs) and secondary metabolites via EVs represents a promising, yet largely unexplored, mechanism that could provide plants with spatial and temporal precision in influencing key microbial processes such as nitrification, denitrification, and nitrogen fixation (**Figures 1A, B**).

Beyond their intracellular roles, several miRNAs such as miR399 and miR2111 function as mobile long-distance signals that move between shoots and roots, and even between neighboring plants. These signals regulate nodulation and phosphate uptake, and act systemically through the phloem (Yang et al., 2024). Their mobility strongly suggests that EVs could act as carriers of these regulatory RNAs across cell boundaries and possibly into the rhizosphere, enabling precise and dynamic regulation of microbial nitrogen-transforming activity (Yang et al., 2024).

Several key miRNAs—including miR160, miR164, miR167, miR169, miR393, miR395, miR399, and miR827—coordinate plant responses to nitrogen and phosphorus limitation by targeting transcription factors, nutrient transporters, and hormone signaling components (Nguyen et al., 2015; Islam et al., 2022). These miRNAs regulate processes such as root development, nutrient uptake, and senescence, forming a dynamic regulatory network responsive to environmental conditions. Packaging these regulatory RNAs into EVs could synchronize internal nutrient signaling with microbial modulation in the rhizosphere, providing plants with a powerful tool to dynamically shape microbial activity.

Future research should focus on quantifying the actual contribution of EVs to plant–microbe communication, clarifying how EV-mediated delivery interacts with other transport pathways, and identifying plant sRNAs that directly target microbial genes involved in nitrogen cycling. Importantly, several miRNAs—including miR165, miR167, miR319, miR396, miR399, and miR827—have also been shown to be differentially regulated under drought and combined nitrogen deficiency and drought stress, with distinct early (7-day) and late (14-day) expression responses (Yaşar et al., 2024). This temporal plasticity suggests that plants dynamically fine-tune their regulatory miRNA expression based on stress type and duration.

Emerging evidence from tomato fruit tissue provides indirect but compelling support for this hypothesis. Under salt, drought, and nitrogen deficiency stress, tomato plants not only increased EV secretion but also selectively modulated the miRNA composition, enriching miR162 and miR1919 while depleting miR9476 (Huang et al., 2025). These shifts were stress-specific and functionally consequential: changes in EV cargo directly altered zinc transporter gene expression and zinc accumulation in intestinal cells. Although derived from fruit tissues rather than root exudates, these finding strongly suggest that plants can reprogram EV composition in response to abiotic stress—raising the possibility that similar mechanisms operate in root-derived EVs to modulate microbial activity in the rhizosphere.

If these regulatory miRNAs are indeed packaged into EVs, plants may be capable of time-sensitive modulation of microbial activity in the rhizosphere, adapting their influence in response to both immediate and prolonged environmental challenges. To test these hypotheses, several methodological and analytical advances are critical—particularly in isolating EVs from soil environments, characterizing their cargo, and linking these molecules to functional outcomes in microbial communities. Complementary approaches, including metagenomics and transcriptomics, can help identify crop genotypes naturally predisposed to associate with microbial consortia that enhance nitrogen-use efficiency.

In parallel, EV research in plants, particularly their role in the rhizosphere, faces significant bioinformatics and data integration challenges. Isolating and characterizing EVs from complex soil environments is a major hurdle, as plant-derived EVs must be distinguished from microbial and abiotic particles. Once isolated, EVs contain a highly diverse cargo—including proteins, lipids, metabolites, and RNAs—which requires the application of multiple omics approaches such as proteomics, metabolomics, and small RNA sequencing. Integrating these heterogeneous datasets to infer the functional role of EVs is particularly challenging, especially when attempting to correlate specific cargo molecules (e.g., miRNAs or secondary metabolites) with microbial community responses.

The spatial and temporal dynamics of EV secretion under changing environmental conditions further complicate this task. In this context, multivariate techniques that explore the latent space—when coupled with mechanistic knowledge of molecular pathways and cellular processes—could significantly enhance data integration and functional interpretation. However, advancing this field also requires the development of new methodologies to investigate and quantify cell-to-cell communication and vesicle-to-cell signaling between plants and the rhizosphere microbiome, which remain poorly understood.

Addressing these challenges could provide the foundation for advances in application. Altogether, integrating these insights could enable precision manipulation of plant–microbe interactions through targeted EV secretion and cargo composition. Such advances may pave the way for breeding crops that actively shape their microbial environment, achieving higher NUE, reduced fertilizer dependency, and lower greenhouse gas emissions—ultimately contributing to climate-smart and sustainable agriculture.
